# Middle Ear Cavity Morphology Is Consistent with an Aquatic Origin for Testudines

**DOI:** 10.1371/journal.pone.0054086

**Published:** 2013-01-14

**Authors:** Katie L. Willis, Jakob Christensen-Dalsgaard, Darlene R. Ketten, Catherine E. Carr

**Affiliations:** 1 Department of Biology, University of Maryland, College Park, Maryland, United States of America; 2 Institute of Biology, University of Southern Denmark, Odense M, Denmark; 3 Department of Otology and Laryngology, Harvard Medical School, Biology Department, Woods Hole Oceanographic Institution, Woods Hole, Massachusetts, United States of America; 4 Department of Biology, University of Maryland, College Park, Maryland, United States of America; University of Lethbridge, Canada

## Abstract

The position of testudines in vertebrate phylogeny is being re-evaluated. At present, testudine morphological and molecular data conflict when reconstructing phylogenetic relationships. Complicating matters, the ecological niche of stem testudines is ambiguous. To understand how turtles have evolved to hear in different environments, we examined middle ear morphology and scaling in most extant families, as well as some extinct species, using 3-dimensional reconstructions from micro magnetic resonance (MR) and submillimeter computed tomography (CT) scans. All families of testudines exhibited a similar shape of the bony structure of the middle ear cavity, with the tympanic disk located on the rostrolateral edge of the cavity. Sea Turtles have additional soft tissue that fills the middle ear cavity to varying degrees. When the middle ear cavity is modeled as an air-filled sphere of the same volume resonating in an underwater sound field, the calculated resonances for the volumes of the middle ear cavities largely fell within testudine hearing ranges. Although there were some differences in morphology, there were no statistically significant differences in the scaling of the volume of the bony middle ear cavity with head size among groups when categorized by phylogeny and ecology. Because the cavity is predicted to resonate underwater within the testudine hearing range, the data support the hypothesis of an aquatic origin for testudines, and function of the middle ear cavity in underwater sound detection.

## Introduction

Vocalizations indicate that hearing has behavioral importance for Testudines [1]. Sea turtles vocalize in air with “ [a] mercy cry and roars and grunts of anger” [2]. Many species of tortoise vocalize in air, most often in the context of mating or distress, including *Gopherus agassizzi, Geochelone carbonaria, Geochelone travancorica, Geochelone gigantea,* and *Platysternon megacephalum* [2]–[4]. Calls of *G. agassizzi* range from 500 to 1000 Hz [3]. Campbell and Evans characterized one of these calls as a possible distress signal because this particular animal was attempting to escape [3]. In one recorded instance, the male of a pair of *G. carbonaria*, vocalized in air while he attempted to mount the female. The vocalization is a “cluck” that is paired with head-bobbing behavior. The authors speculate that it is similar to the attraction calls observed in other species, which are used both in mating and in parent-offspring interactions [1], [3]. Campbell and Evans further characterize the vocalization of *G. carbonaria* [3]. The cluck previously described was in the range of 500–2500 Hz. Playbacks of “cluck” recordings elicit head movements. *G. travancorica* is, thus far, the only tortoise species that is known to call in chorus [2]. These vocalizations had the most energy from 1700–2000 Hz. *P. megacephalum* produces a two-part call with frequency components from 500–4000 Hz. Campbell and Evans observed this particular type of vocalization only in juveniles. Aside from these studies, little to nothing is known about the behavioral and social relevance of any testudine vocalizations.

The best candidate species for investigations of the behavioral relevance of vocalization among the Testudines is *Chelodina oblonga* (Oblong Turtle or Snake-necked Turtle), which exhibits an extensive vocal repertoire that can be divided into 17 categories, including both percussive and complex vocalizations [5]. Animals of different ages and both sexes were recorded vocalizing in air and underwater. These vocalizations range in frequency from 100 Hz to over 20,000 Hz, a much greater range than is found on other previously studied species. Despite this wide range, the calls are almost all under 4 kHz. The frequency spectra are also quite varied, from harmonic to noisy. This species inhabits turbid water, thus decreasing its ability to use visual cues [5]. This is would suggest a reliance on non-visual cues. The spectra covered by these calls do not necessarily imply that the animal can hear the calls throughout the entire range. Birds do not hear the entire spectra of their song [6]. Neither the anatomical structures involved in vocalization nor their hearing thresholds have yet been described for *C. Oblonga*.

Given this evidence for middle- and high-frequency vocalizations, it is possible that some pleurodires (side-necked turtles), including *C. oblonga*, may hear above 2 kHz, i.e. above reported hearing thresholds [5]. Taking new findings about vocalizations into account, the idea that turtles are relatively insensitive to sound should be reconsidered. If vocalizations are important for mating or other social interactions, there would be selective pressure for auditory acuity. Given that multiple species vocalize, some at frequencies higher than previously measured, hearing in these species should be more fully investigated.

Testudines are divided into two suborders: Cryptodira and Pleurodira. Extant cryptodires include three superfamilies: Chelonioidea, Testudinoidea, and Trionychoidea. Pleurodires, (Side-necked Turtles) include the superfamily Pelomedusoidea and the family Chelidae. Testudines, while monophyletic, have adapted to a wide variety of ecological niches and lifestyles [7]. Ecologies range from marine (Sea Turtles) to semi-arid desert biomes (Tortoises). Sound transmission, production, and reception are affected by the medium in which the animal lives and communicates. Environmental sounds, as well as those generated by predators, prey, and conspecifics provide essential information.

Multiple skull bones comprise the middle ear cavity [8]. As in other tetrapods, the inner ear is encased by the cavum labyrinthicum. The interior of the middle ear cavity is called the cavum tympani, which is formed from the quadrate and the squamosal. The middle ear is bordered anterolaterally and dorsally by the quadrate, dorsally by the opisthotic, medially by the prootic and opisthotic, and ventrally by pterygoid. The columella extends from the oval window, where it forms the stapedial footplate [9], through the cavum acustico-jugulare and incisura columellae auris, into the middle ear cavity. The columella is the primary transducer of sound as demonstrated by Wever and Vernon who showed that the hearing capability of an animal was greatly reduced after the columella was clipped [10]. The columella terminates on the extracolumella via a short, hinged joint [10], [11]. The extracolumella is cartilaginous and forms the tympanic disk.

**Figure 1:**
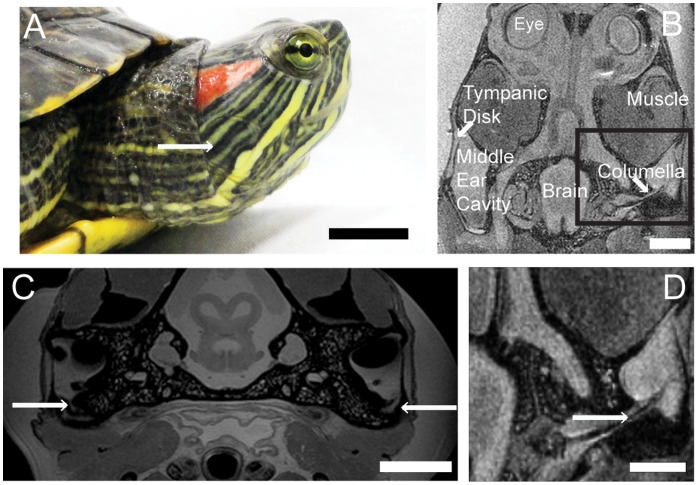
Anatomical structures of the testudine audiotory system in *Trachemys scripta elegans*. A. Lateral view of head (1 cm scale bar). B: Horizontal MR image. (500 mm scale bar) C: Transverse MRI at the level of the tectum. Arrows indicates Eustachian tubes (500 mm scale bar). “Muscle” is the splenius capitus. D: Horizontal MR image, enlarged from box in B. The columella runs through the middle ear cavity to the inner ear. Arrow indicates the columella (500 mm scale bar).

In *Trachemys scripta elegans* (Red-eared Slider turtle), the tympanic disk is about 0.5 mm thick [10], [11]. The tympanic disk is visible on the animal through the relatively undifferentiated skin (Fig. 1), which adheres to the tympanic disk by a thin layer of connective tissue [9], [11]. The tympanic disk moves via a hinged connection to the bony capsule wall surrounding it [10], [11]. The disk is primary sound receiving structure of the turtle ear [9]–[12] (Fig. 1). Behind the tympanic disk is the middle ear cavity. Laser vibrometry measurements suggest that the air in the middle ear cavity resonates in the underwater sound field, driving the tympanic disk [11], Comparisons of hearing in air and under water in *Trachemys scripta elegans* show these turtles are more sensitive to sound under water [11].

These findings raise many questions. Greater sensitivity to sound under water could be conferred by multiple adaptations. Christensen-Dalsgaard and colleagues suggest that the origin of greater sensitivity to underwater sound is the ability of the middle ear cavity to resonate in the underwater sound field, increasing sensitivity at resonant frequencies [11]. Is this type of middle ear cavity is a feature of all turtles and tortoises, or is it only found in those testudines that spend significant time underwater? How do variations in middle ear structures inform our understanding of the evolutionary history of testudines? We demonstrate here that middle ear scaling and morphology is similar across extant species, regardless of ecological niche or phylogenetic position.

## Results

### Anatomy

**Figure 2:**
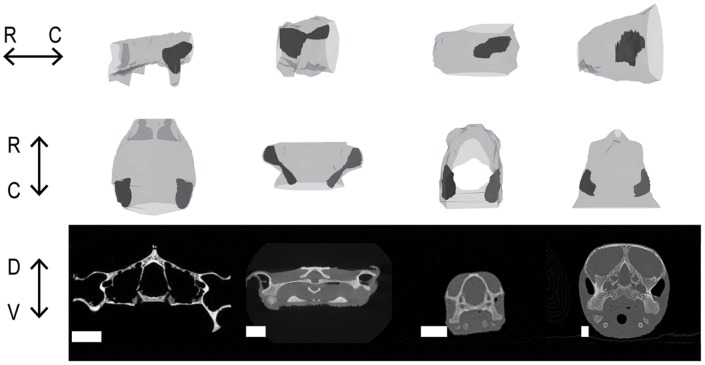
Examples of middle ear morphology of extant turtles and tortoises. Middle ear cavities are in black with skulls in gray. Top row. Lateral view of the left side. Middle row: Dorsal view. Bottom row: Cross section CT images at the level of the middle ear cavity. Species in columns from left to right: *Gopherus polyphemus, Chelus fimbriatus, Trachemys scripta elgans, Lepidochelys kempii*. Scale bars = 1 cm. R = rostral. C = caudal. D = dorsal. V = ventral. Note that *G. Polyphemus* was scanned as only a skull.

In all species examined, the Eustachian tubes were small and opened adjacent to the tympanic disk on the ventral wall of the middle ear cavity, connecting the cavity to the pharynx (Fig. 1) [10]. We used *Trachemys scripta elegans* as an example species for some more detailed anatomical studies. In *T. scripta elegans*, the Eustachian tubes are narrow but detectable on MR images (Fig. 1 C). The fluid-filled tube appeared as a grey duct, because the middle ears were filled with saline postmortem to optimize the image. At the opening of the Eustachian tube from the middle ear, on one sample of *T. scripta elegans*, the tube measured about 500 µm in diameter. All species examined had middle ear cavities in the general form of paraboloids with the long axis oriented rostrocaudally, parallel to the midline (Fig. 2).

### Allometry of Middle Ear Cavity Volume in Trachemys Scripta Elegans

**Figure 3:**
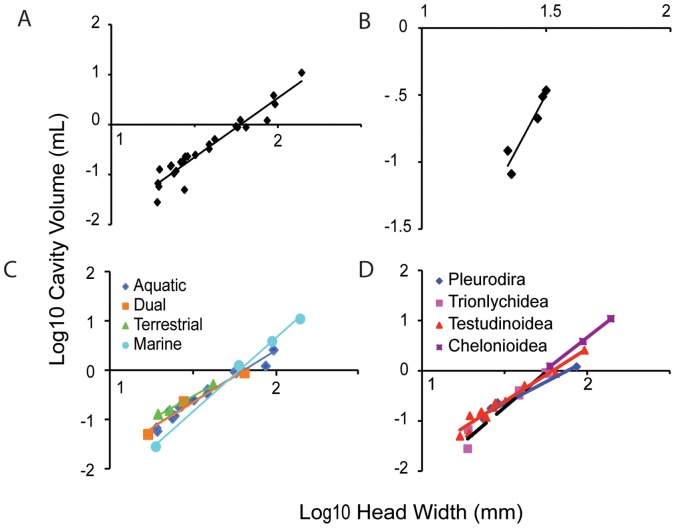
Allometry of middle ear cavities. A: Scaling of middle ear cavity volume and head width across extant testudines B: Scaling of volume and head width in *Trachemys scripta elegans*. C: Scaling of middle ear cavity volume and head width across extant testudines divided by ecological niche. D: Scaling of middle ear cavity volume and head width across extant testudines divided by phylogenetic position.

In order to assess changes over the lifespan of an animal, an allometric series of 5 Red-eared Sliders (*T. scripta elegans*) was analyzed separately from the other species [13] (Fig. 3 B) and included in the whole data set (Fig. 3 A). For *T. scripta elegans*,
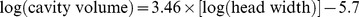
with r^2^ = 0.89 showing that, during the growth of an animal, head size increases allometrically with body size. From visual inspection, the overall shape of the cavity did not change with the body size.

### Cross-species Comparisons

In the 25 species from 12 families examined (Table 1), the middle ear cavity was a paraboloid (Fig. 2) that scaled with head size (Fig. 3). The scaling followed the equation
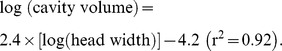



**Table 1:** Phylogenetic relationships of the species studied.

Suborder	Superfamily	Family	Subfamily	Species	Ecology
Pleurodira		Chelidae		*Elseya dentata*	Aquatic
				*Chelus fimbriatus*	Aquatic
	Pelomedusoidea	Podocnemididae		*Pelusios sinuatus*	Aquatic
				*Podocnemis unifilis*	Aquatic
Cryptodira	Trionychidea	Carettochelyidae		*Carettochelys insculpta*	Aquatic
		Dermochelyidae		*Dermochelys coriacea*	Marine
		Kinosternidae	Staurotypinae	*Staurotypus salvinii*	Aquatic
			Kinosterninae	*Kinosternon bauri*	Aquatic
		Trionychidae	Trionychinae	*Trionyx triunguis*	Aquatic
				*Apalone mutica*	Aquatic
	Testudinoidea	Platysternidae		*Platysternon megacephalum*	Dual
		Bataguridae	Geoemydinae	*Rhinoclemmys pulcherrima*	Terrestrial
				*Cuora amboinensis*	Dual
		Emydidae	Emydinae	*Glyptemys (Clemmys) muhlenbergii*	Dual
				*Emys orbicularia*	Aquatic
				*Malaclemys terrapin*	Aquatic
			Deirochelyinae	*Trachemys (Pseudemys) scripta elegans*	Aquatic
				*Chrysemys picta picta*	Aquatic
		Testudinidae (Tortoises)		*Testudo horsfieldi*	Terrestrial
				*Gopherus polyphemus*	Terrestrial
		Chelydridae		*Chelydra serpentina*	Aquatic
				*Macroclemys temminckii*	Aquatic
	Chelonioidea (Sea Turtles)	Cheloniidae		*Carretta caretta*	Marine
				*Chelonia mydas*	Marine
				*Lepidochelys kempii*	Marine

At least one representative from each family of testudines was included in this study, with the exception of the Dermatemydidae, a monotypic family containing *Dermatemys mawii* for which no museum specimen was available.

The exception to this morphology was the Matamata, *Chelus fimbriatus*, which has a hyperboloid (hourglass-shaped) middle ear cavity, which also scaled following the above equation. *C. fimbriatus*, is a pleurodire (Side-necked Turtle) and inhabits the Amazonian river basin. Its skull is dorsoventrally flattened, and its unusual skull morphology may constrain middle ear cavity dimensions.

The wavelengths of the sound range in question are much greater than the dimensions of the cavity and thus the effects of the shape of the cavity are negligible [14]. Because the volume of the cavity is the primary factor for acoustic characteristics of the middle ear cavity at frequencies relevant to testudines, we used a sphere equal to the measured paraboliod volume for resonance calculations for each middle ear cavity [14].

### Scaling and Morphology do not Change with Ecology or Phylogeny

We compared the scaling of the middle ear cavity volumes, with head width as a covariate, among the ecological groups, using univariate ANOVA, and found no significant differences (p = 0.494, model 2 regression r^2^ = 0.942). When the scaling of the middle ear cavity volumes with head width was compared among the phylogenetic groups by using univariate ANOVA, no significant differences were found (p = 0.282, model 2 regression r^2^ = 0.773).

### Middle Ear Cavity can Function as a Resonator

**Figure 4:**
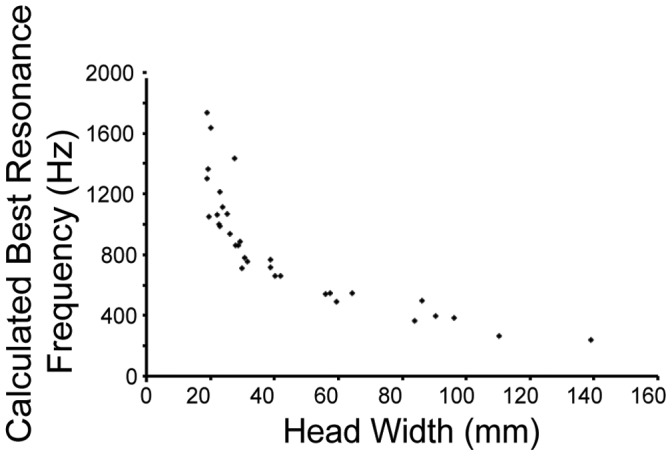
Calculated best resonance underwater frequency of middle ear cavities of extant species, changing with head size.

Calculations were performed for a model of an air-filled sphere vibrating in an underwater sound field [14]. Unlike the ears of lepidosaurs and archosaurs, testudine ears are not acoustically coupled [11] and because the wavelengths are large compared with the size of the cavity, calculations were based only on the volume of the middle ear cavity. Middle ear cavities ranged in volume from 0.03 mL to 10.9 mL; head widths ranged from 19–140 mm (Fig. 3). By modeling the middle ear cavity as a sphere vibrating underwater, we calculated the resonance frequencies of the cavities as ranging from 240–1740 Hz (Fig. 4).

### Sea Turtles (Family Cheloniidae)

Sea Turtle middle ear cavities contain varying amounts of fatty tissue adjacent to the tympanic disk, even differing bilaterally within the same animal [9], [15]. The amount of fatty connective tissue, and therefore the amount of residual air space in the middle ear, varied among the Sea Turtles examined, which complicated resonant frequency calculations. Because it was unclear what the exact volume of the middle ear fats might be and to what extent they compress with depth, our calculated resonance frequencies might be lower than the actual resonance frequencies experienced by the sea turtles (smaller effective resonating volume results in higher resonance frequencies). However, to date there are no published measurements of the maximal or minimal volumes for these fats nor of their elasticity or compressibility. Scans of both live and post-mortem sea turtle specimens demonstrate that the space occupied by soft tissue in the middle ear cavity can vary between individuals and even bilaterally within the same turtle, but it is not known whether these variations remain underwater. In the absence of such data, we calculated the maximal cavity volume based on skull morphology. Based on the skull structure, the allometry of the middle ear cavity of sea turtles did not scale differently from the other testudines (Fig. 3 C, D).

### Extinct Species

**Figure 5:**
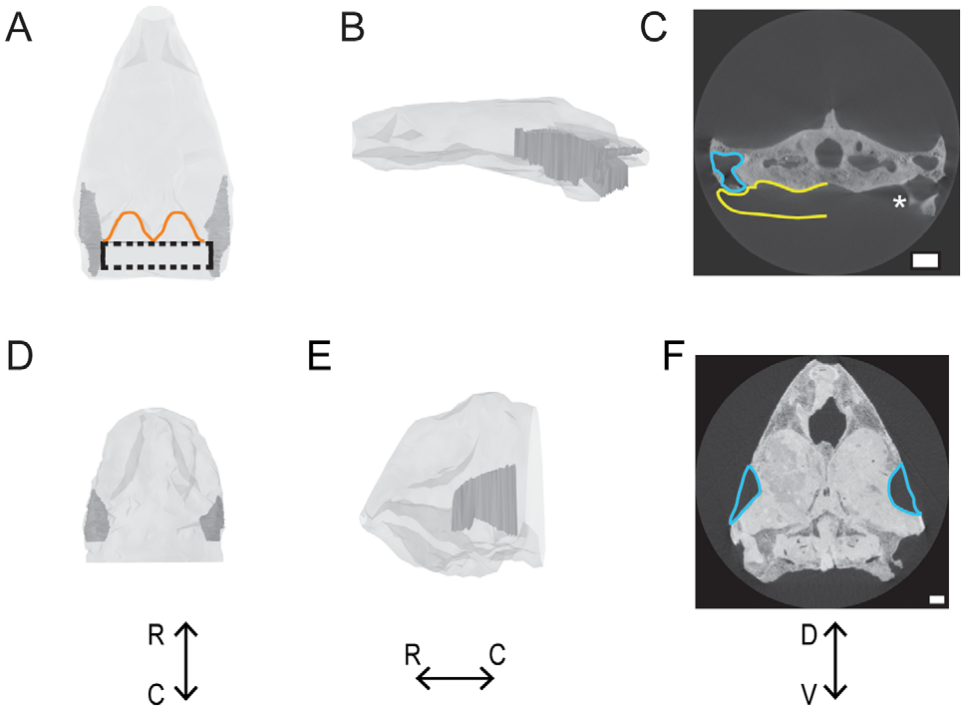
Examples of middle ear cavities of extinct testudines. A-C: Connected ears of *Galianemys emringeri.* Connected middle ears are shown in dark gray; the skull is shown in light gray. The maximum space that the connected middle ears could possibly occupy is indicated by the dashed line. The dorsocaudal edge of the skull is outlined in orange. D-F: Separated ears of *Nicholsemys baieri.* Isolated middle ears are show in dark gray; skull is shown in light gray. A & D: Dorsal view. B & E: left lateral view. C & F: Transverse view from CT. Middle ear cavities are outlined in blue, and possible extent of middle ear cavity into pharynx is yellow. Asterisk indicates most caudal part of the middle ear cavity that can be seen intact before it opens into the pharynx. Scale bars = 1 cm. R = rostral. C = caudal. D = dorsal. V = ventral.

CT scans of several extinct species, including *Galianemys emringeri, Galianemys whitei, Nichollsemys baieri,* and *Hamadachelys escuilliei*, revealed that Galianemys and Hamadachelys species have middle ears that are connected through the mouth, to the extent observable from the fossilized remains (Fig. 5), while *Nichollsemys baieri* has more isolated ears, like the extant testudines (Fig. 2). In the CT images of the Galianemys and Hamadachelys species, there is a clear opening from the middle ear cavity into the mouth (Fig. 5 C). This large opening is not seen in *N. baieri.* As the Eustachian tubes are comprised of soft tissue, the size of the Eustachian tubes could not be determined.

Connected ears were also shown in *Proganochelys* [16]. These specimens were not reconstructed in detail, nor used for volume calculations, because of the potential distortions derived from fossil compression. *Galianemys emringeri, Galianemys whitei, Nichollsemys baieri* were pleurodires, and *Hamadachelys escuilliei* a cryptodire. All of the specimens were found in Cretaceous formations.

## Discussion

### Middle Ear Cavities Enhance Hearing

Resonance via enlarged middle ear cavities has been shown to affect hearing in a number of vertebrate classes, both in air and under water. For example, the enlarged middle ear cavity of kangaroo rats underlies good hearing thresholds below 3 kHz, particularly in the 1–2 kHz range [17], [18]. Similarly, the bulla (middle ear cavity) in gerbillines acts like a Helmholtz resonator, lowering hearing thresholds [19]. One example of air-filled structures lowering hearing thresholds underwater is Ostariophysan fish, which couple swimbladders to Weberian ossicles, enabling sound pressure hearing, not just detection of particle motion [20]–[23]. Similarly, the ranid frog *Lithobates* (*Rana*) *catesbeiana* is more sensitive to sound below 200 Hz underwater than in air and is equally sensitive in air and in water for frequencies above 400 Hz, possibly due to specialization of the amphibian papilla [24]. The middle ear cavity of the African clawed frog (*Xenopus laevis*) provides hearing advantages underwater [25]. The ear of *Xenopus* works like the turtle ear, with cartilaginous tympanic disks and an air-filled resonating cavity. *Xenopus* also has further adaptations for underwater hearing, including a tighter coupling and lower lever ratio between the tympanic disk and ossicles than do the ranid frogs [26].

Wever and Vernon were aware of the potential for middle ear resonance in their studies of turtle hearing [10]. They calculated resonance frequencies for the middle ear cavities in *Chrysemys picta picta* and *Trachemys* (*Pseudemys) scripta* in air to be 6 kHz by using a closed tube model where the resonance frequency quarter wavelength matches the length of the tube. Volumes used in obtaining this value were not published. Because 6 kHz was well above measured highest audible frequency (about 2 kHz), they discounted any increased sensitivity modeling based on resonance. Recent studies, however, show that the ear of *Trachemys scripta elegans* is more sensitive to sound underwater than in air [11], where resonance frequencies are much lower. We hypothesize that the conserved structure of the testudine ear is an adaption for underwater hearing that was retained by neutral selection.

Middle ear cavities are also interesting from the perspective of understanding how a major vertebrate group processes sound. Hearing has been documented in multiple species of testudines, demonstrating that these animals have auditory sensitivity, albeit with higher thresholds in air than those of other reptiles [9]. Six testudine species have published in air audiograms (Table 2), with best hearing frequencies below 1000 Hz (around 400–600 Hz). There is much to be learned about how the testudine middle ear responds to sound underwater. Laser vibrometry studies, perhaps from post-mortem samples from a variety of species, could be used to test the hypothesis that both turtle and tortoise ears would respond well to underwater sound. The fossil specimens without isolated middle ear cavities could represent either the ancestral diapsid condition, or a secondary loss. As more extinct species are discovered, answers to this question should become clearer.

**Table 2:** Published testudine in-air audiograms.

Species	Lowest TestedFrequency (Hz)	Highest TestedFrequency (Hz)	Best FrequencyRange (Hz)	Reference
*Chelonia mydas*	30–40	2000	300–400	[15]
*Clemmys insculpta*	100	5000	500	[48]
*Chrysemys picta picta*	100	4000	400–500	[48]
*Caretta caretta*	250	1000	250–500	[49]
*Terrapene carolina carolina*	30	4000	400	[50]
*Trachemys scripta elegans*	100	3000	500	[48]
*Trachyemys scripta elegans*	64	1000	400–700	[51]
*Trachemys scripta elegans*	100	1000	400–500	[11]

### Sea Turtle Ears

The function of the fatty tissue in Sea Turtle middle ears is unknown, while the high degree of variability in these structures adds to the mystery. There are a variety of hypotheses about their function, including their being an adaptation to the pressure resulting from deep diving [9], [15], or a secondary pathway for sound transmission, in a manner analogous to the fatty channels in the jaws of marine mammals [27]. While our data do not address the function of this tissue, they do suggest that fatty tissue in the middle ear may be secondary adaptation in Sea Turtles, because their skull elements and allometry are the same as the other testudines.

### Phylogenetic Position of Testudines

As shown by Christensen-Dalsgaard and colleagues, at least one species of turtle hears well under water than in air, largely due to the middle ear cavity [11]. Given that the middle ear cavity resonates underwater within the published in-air testudine hearing range and that the middle ear cavity resonates beyond that range in air [10], our findings of unchanging middle ear cavity allometry among the testudines support the hypothesis of an aquatic origin for this group. Since the tortoises retained this allometric relationship, we further hypothesize that the middle ear cavity does not impede hearing in air.

Analyses of the hearing of testudines have been complicated by their ill-defined relationship to other major vertebrate groups. Since testudines are anapsids, they had been considered an extant representative of the parareptiles, which places them as a sister to the entire diapsid clade. This position was supported by some morphological analyses [28], [29]. Rieppel and deBraga, however, proposed that testudines were the sister group to lepidosaurs [30]. They state that the traditional view, in which the number of temporal fenestra is the deciding factor for determining vertebrate relationships, is too narrow. Their analyses included a much wider range of non-skull characters [30]. A recent study of mesosaurid skulls supports diapsid affinities of the testudines [31]. Interestingly, data that support testudines being either the sister group to the archosaurs or to the entire diapsid clade support a terrestrial origin of testudines [29]; conversely, the data that support testudines being the sister group to lepidosaurs support an aquatic origin [32].

The advent of molecular techniques and the application of these methods to phylogenetic problems called into question the traditional understanding of the position of testudines. Phylogenomic analyses have led to a reevaluation of the position of the testudines. These studies robustly support the position of testudines as sister to the archosaurs, with the archosaurs remaining monophyletic [33], [34]. Hedges and Poling found that in all but one gene, testudines were most closely related to archosaurs [35]. The position of testudines within the diapsid clade has been supported by other molecular analyses [36]–[39].

**Figure 6:**
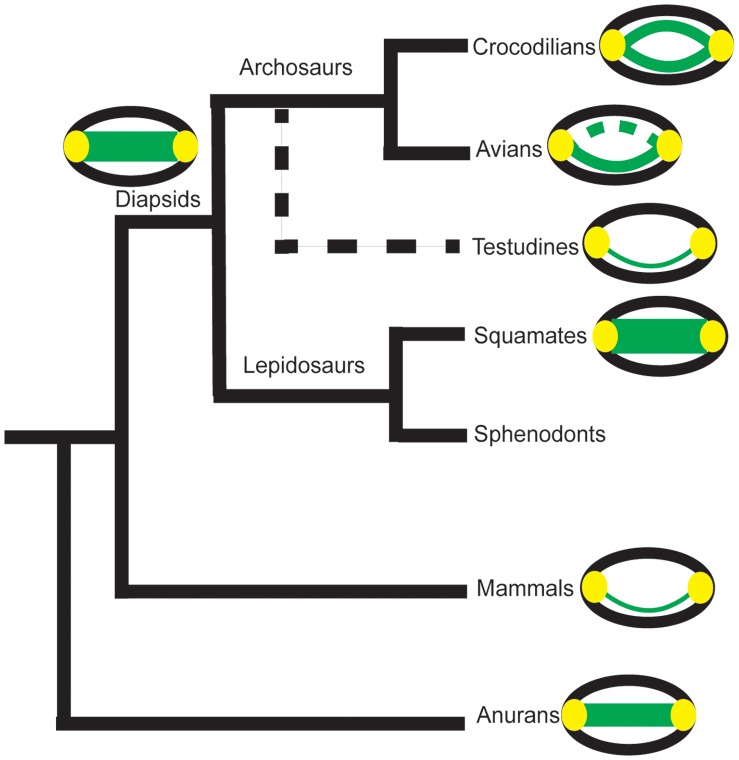
Proposed middle ear structure across some extant vertebrate taxa. Skulls are shown in black, tympanic ears in yellow, connections between the ears (Eustachian tubes or through the buccal cavity) in green. The dashed line on the avian diagram indicates trabeculated bone. The proposed diapsid ancestral condition is also shown. The dashed branch to testudines indicates their suggested phylogenetic position [33]–[35].

While our data do not directly address the phylogenetic position of testudines, they support an aquatic origin for this group. There is also support for this claim from the fossil record: *Odontochelys*, the most basal testudine discovered thus far, appears to have been aquatic [40]. It is parsimonious to assume that the common ancestor of archosaurs, lepidosaurs, and testudines had coupled ears that opened into the pharynx, since coupled ears are the ancestral condition for tympanic ears (Fig. 6) [41], [42]. Our data suggest that Testudines secondarily evolved acoustically isolated middle ear cavities because of the improved underwater sound sensitivity they provide.

## Methods

### Imaging

We examined the middle ear cavity and associated structures using X-ray computed tomography (CT) and magnetic resonance imaging (MRI) (Table 1). Specimens (*Trachemys scripta elegans* and *Macroclemys temminckii*) were prepared for magnetic resonance (MR) scanning by euthanasia via an overdose of Euthasol (Virbic Animal Health, Fort Worth, TX). The heads were then removed and immersion-fixed in 4% paraformaldehyde (PFA) in 0.01 M phosphate buffered saline (PBS) for a minimum of 1 week. The fixed heads were rehydrated in 0.01 M PBS a minimum of 24 hours before the scan. In order to optimize the image the middle ear cavities were filled with PBS: one syringe was inserted into the tympanic disk to remove air while another syringe was simultaneously used to inject 0.01 M PBS.

*Trachemys scripta elegans* was chosen as an example species for an allometric series because it is an amphibious invasive species and commercially available. Animals were obtained from a commercial dealer. Furthermore, the small head size allowed imaging in the most powerful MR scanner (9.4 T). MR images of *Macroclemys temminckii* and *T. scripta elegans* were acquired at the Armed Forces Institute of Pathology (Rockville, MD). Prior to imaging, larger heads were sealed in a plastic bag filled with 0.01 M PBS and imaged with a 72 mm volume coil on a Bruker Biospec spectrometer (Bruker Biospin, Inc. Billerica, MA) coupled to a horizontal-bore magnet (diameter: 20 cm) operating at 7 T (300 MHz for protons) using a Rapid Acquisition with Relaxation Enhancement (RARE) sequence with the following acquisition parameters: TR/TE = 1500/10 ms, NA = 4, RARE = 8. Small heads were immobilized in glass tubes (o.d. 25 mm) filled with PBS and imaged with a 25 mm RF insert on a Bruker DMX spectrometer (Bruker Biospin) coupled to a wide-bore magnet (dia. 89 mm) operating at 9.4 T (400.13 MHz for protons). Typical RARE images had a voxel resolution of 100×100×100 µm,) and the analyses were performed using 512 matrix TIFF images.

For all marine species, as well as *Trachemys scripta elegans* and *Malaclemmys terrapin*, submillimeter, ultrahigh resolution computerized tomography (UHRCT) images were obtained on a Siemens Volume Zoom CT scanner at the Woods Hole Oceanographic Institution Imaging Facility. Marine species were obtained post-mortem after death by natural causes. A spiral protocol was employed with 120 kV, 100 mA, 150 effective mAS, 0.5 mm collimation, 0.5 mm/sec table feeds and a 0.5 mm table pitch. Both live (physically restrained) and post-mortem turtles were scanned prone, head first, with scans acquired in the transaxial (shorter cross-section) plane. Images were reconstructed using soft, ultra-high bone, and lung kernels at 0.1 and 0.5 mm increments for the whole head and data based magnifications at smaller FOV of the ear regions alone. The 0.1 mm images provided image data sets with isotropic 100 µm voxel resolution, which were used for volume measurements and cavity reconstructions in 3D. Raw attenuation data and all 512 matrix DICOM images were archived onto CD and magneto-optical disks. In each of these programs, tissues were selected for auto-segmentation based on Hounsfield Unit values for tissue attenuations and air space attenuation. The auto-segmentations were reviewed visually and segmentation boundaries corrected when they incorporated inappropriate adjacent regions.

For all other species, CT images were obtained from DigiMorph (University of Texas, Austin). The images were 1024×1024 16-bit TIFF format. Scan parameters varied some depending on the specimen. A typically example follows: P250D, 420 kV, 1.8 mA, one brass filter, empty container wedge, 190% offset, integration time of 64 ms, slice thickness was 0.5 mm, S.O.D. was 698 mm, 1400 views, one ray averaged per view, one sample per view, interslice spacing of 0.4 mm, field of reconstruction of 268 mm (maximum field of view 280.1441), reconstruction offset of 6100, reconstruction scale of 3200. Ring-removal processing was based on correction of raw sinogram data using IDL routine “RK_SinoRingProcSimul” with parameter “BESTOF5.” This is a standardized process done for all CT scans by the imaging facilties. For an overview of the analysis of CT images, see [43]. The extinct species used were *Galianemys emringeri* (sample ID: AMNH 30035), *Galianemys whitei* (sample ID: AMNH 29987), *Nichollsemys baieri* (sample ID: TMP 97.99.1), and *Hamadachelys escuilliei* (sample ID: MDE-T-03).

### Analysis

All scan files were converted to TIFF stacks and imported into Neurolucida (MicroBrightField Bioscience, Williston, VT). For species that were scanned using both MR and CT, all data sets were used. The outlines of the structures were all traced manually in serial sections. In CT scans, the lateral edge of the middle ear cavity was defined by connecting the most medial points of bone in images where the cavity was open with a straight line. In images where the soft tissue was visible, that line was drawn through the middle of the tympanic disk. Since some the CT images usually did not include the soft tissue tympanic disk, a straight line across the opening was the best approximation. These tracings were analyzed using the NeuroExplorer module to calculate the enclosed volume. Reconstructed area is accurate to one micrometer (MicroBrightField stated accuracy). Head widths were measured as a straight line across the widest part of the head, accurate to 0.1 micrometer. Approximate head widths were confirmed as the same with calipers when possible.

Resonance was calculated by modeling the middle ear cavity as an air-filled sphere vibrating underwater using the following equation:
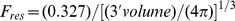
(frequency in Hertz) [14]. Because the frequencies in question are low, and therefore the wavelengths much larger that the dimension of the cavity, the cavity can be treated as a lumped element with a resonance frequency that only depends on volume.

Univariate ANOVA tests were performed with the middle ear cavity volume co-varying with head width data categorized by ecological niche and phylogenetic position (Table 1). Ecological niche was defined by the medium in which the species spends the majority of its life. We divided the non-marine species according to how much time they spent in the water, in order to perform a univariate ANOVA test among the ecological niches. Animals that spent the majority (greater than 60%) of their time in non-marine environments (e.g. pond turtles) were categorized as aquatic. Sea turtles were categorized as marine. Animals spending the majority of their time on land (e.g. tortoises) were categorized as terrestrial. Those species spending approximately equal amounts of time on land and water were categorized as “dual”. We divided the Crypotodirae into superfamilies (Trionychidea, Testuinoidea, Chelonioidea), in order to perform a univariate ANOVA test among the phylogenetic groups. Phylogentic position was determined according to the species information from the University of Michigan Museum of Zoology [44]. Ecological niches were from the descriptions by [45]. We analyzed Pleurodirae as one group because of the small number of species available and because there are far fewer extant species relative to the cryptodires.

Experiments were performed according to the guidelines approved by the Marine Biological Laboratory (Woods Hole, MA, USA), the University of Maryland Institutional Animal Care and Use Committees (IACUC) and the Danish National Animal Experimentation Board (Dyreforsøgstilsynet).

### Conclusions

After separating species by ecology and phylogeny (Fig. 4), there were no significant differences in the variation of middle ear cavity volume and head width, suggesting that there has been little modification among extant testudines. Since middle ear cavities enhance hearing under water [11], it follows that testudines should have lower hearing thresholds in water than in air. A lower hearing threshold under water than in air could only theoretically apply to the terrestrial species. Since not all extant testudines are aquatic or amphibious, the most probable explanation for this constancy is that neutral selection has maintained middle ear cavity scaling.

Given constancy in middle ear cavity scaling, we hypothesize that the most recent common ancestor of the extant testudines was primarily aquatic and had separated middle ears, an assertion supported by two observations from the fossil record. First, in some extinct species of testudines, including *Galianemys emringeri, Galianemys whitei,* and *Hamadachelys escuilliei,* the middle ear cavities opened into the mouth, as does the internally coupled, pressure-difference receiver ear of lizards [24], [46], [47]. It has been argued that coupled ears are both the simplest configuration of, and the ancestral condition for, tympanic ears (Fig. 6) [42]. Second, isolated middle ear cavities appeared in both the extinct marine cryptodire, *Nichollsemys baieri* [48], and independently in the mosasaurs (marine lizards) [49]. The evolution of isolated middle ear cavities in testudines would have provided some selective advantage, which we hypothesize was an increased sensitivity for conspecific vocalizations and auditory scene analysis in a primarily aquatic habit, which may then have been retained by neutral selection.
